# Unusual malposition of peripherally inserted central catheters into the thymic vein and middle thyroid vein: A case series

**DOI:** 10.1097/MD.0000000000042800

**Published:** 2025-06-20

**Authors:** Xuemei Li, Chang Liu, Hongxiu Chen, Zhoupeng Wu, Danmei Liang, Xiaoxia Zhang

**Affiliations:** a West China School of Nursing, Sichuan University, Chengdu, Sichuan Province, P.R. China/Department of Emergency Medicine, West China Hospital, Sichuan University, Chengdu, Sichuan Province, P.R. China; b Disaster Medical Center, Sichuan University, Chengdu, Sichuan Province, P.R. China; c Nursing Key Laboratory of Sichuan Province, West China Hospital, Sichuan University, Chengdu, Sichuan Province, P.R. China; d Department of Radiology, West China Hospital, Sichuan University, Chengdu, Sichuan Province, P.R. China; e Innovation Center of Nursing Research, Nursing Key Laboratory of Sichuan Province, West China Hospital, Sichuan University, Chengdu, Sichuan Province, P.R. China; f West China School of Nursing, Sichuan University, Chengdu, Sichuan Province, P.R. China; g Department of Vascular Surgery, West China Hospital, Sichuan University, Chengdu, Sichuan Province, P.R. China; h Division of Head & Neck Tumor Multimodality Treatment, Cancer Center, West China Hospital, Sichuan University, Chengdu, Sichuan Province, P.R. China.

**Keywords:** catheter malposition, central venous catheter, middle thyroid vein, peripherally inserted central catheter, thymic vein

## Abstract

**Rationale:**

Correct catheter tip placement is the foundation for catheter utilization and the prevention of catheter-related complications. We describe 2 unusual peripherally inserted central catheter (PICC) malpositions in the left thymic vein and the right middle thyroid vein, which are, to our knowledge, the first 2 cases of PICC malposition in these 2 veins.

**Patient concerns:**

Both patients had subtle clinical signs of catheter malposition on initial post-procedure chest X-ray radiography or during catheter duration. However, all signs were overlooked.

**Diagnoses:**

Two catheter malpositions were incidentally discerned and diagnosed using a winding and delayed modality.

**Interventions:**

One of the catheters was removed at a local hospital, the other catheter tip was adjusted to the superior vena cava by the advanced practice nurse.

**Outcomes:**

Two catheter malpositions were incidentally discerned and diagnosed over the course of a winding and delayed procedure, resulting in belated catheter manipulation.

**Lessons:**

Although neither patient experienced significant catheter tip-related events, the delayed diagnostic process led to the presentation of these 2 unusual cases, to guide further recognition of this complication. Some principles should be followed to avoid rare PICC malposition and its fatal complications, including verification of tip before using catheter, use of real-time imaging guidance, regular monitoring of long-term dwelling catheter, and proactive assessment of catheter or direct communication with radiologists particularly in cases where catheter tip malposition is highly suspected.

## 1. Introduction

Peripherally inserted central catheters (PICCs) provide stable venous access to patients for various purposes, such as drug administration, blood sampling, and central venous pressure monitoring. Correct catheter tip placement is fundamental for effective catheter utilization. Real-time imaging-guided catheterization markedly enhances the precision of PICC tip localization.^[1]^ However, in sporadic instances, the catheter may enter an unusual vein and have fatal consequences. This case series delineates 2 delayed-diagnosed unusual PICC malpositions in the left thymic vein and the right middle thyroid vein (RMTV). Although the 2 patients had no obvious catheter tip-related events, the delayed and circuitous process of diagnosis prompted us to present these 2 cases for further recognition of this possible complication and to help medical staff discern it promptly. To our knowledge, these are the first 2 cases of malpositioned PICCs in these 2 veins.

## 2. Case presentation

The study was reviewed and approved by the ethics committees of the West China Hospital, Sichuan University [No. 2024-391]. Written informed consent was obtained from all patients for research purposes. The manuscript has been reported following the CARE guidelines.^[2]^

### 2.1. Patient 1

**Figure 1.**
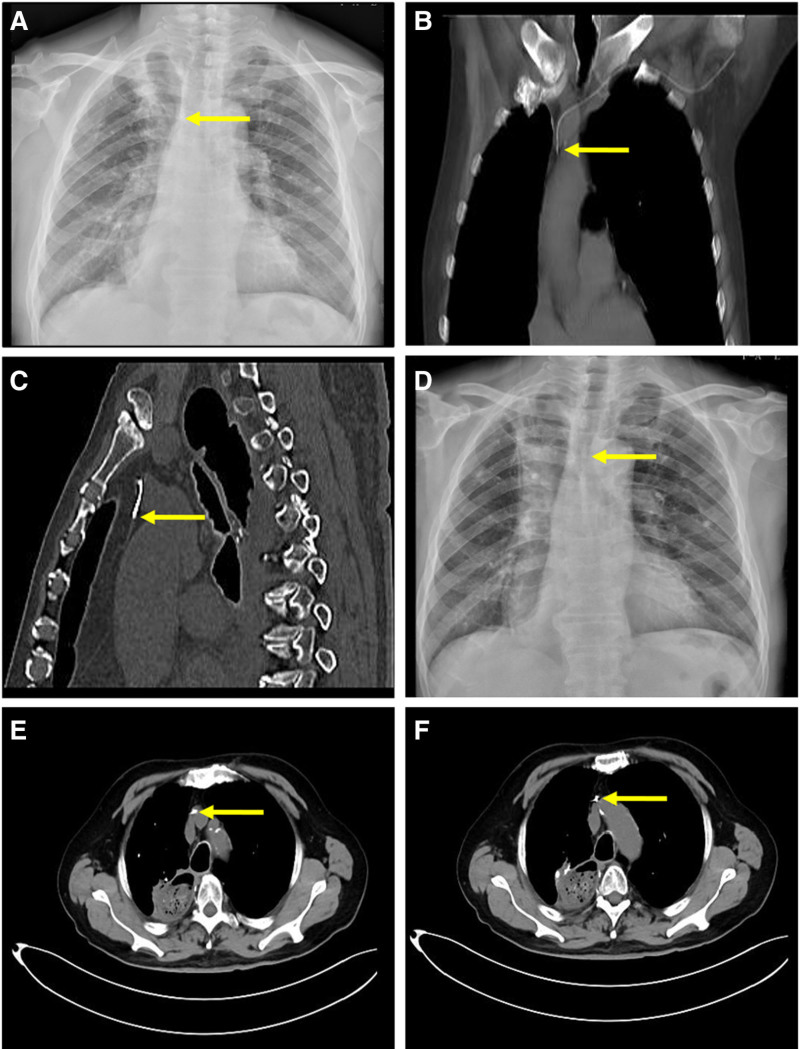
Radiographic information for Patient 1.

A 74-year-old male patient who had been diagnosed with esophageal cancer and pulmonary metastasis was admitted to our oncology center for chemotherapy on March 10, 2023. On March 17, an advanced practice nurse inserted a PICC via the patient’s left basilic vein under local anesthesia and ultrasonography. The procedure was smooth, with good blood return and no resistance during guidewire retraction. After catheterization, an anterior-posterior chest radiograph (AP-CXR) was performed immediately, indicating that the catheter tip was positioned at the sixth thoracic vertebral (T6) level, which was the desired location (Fig. 1A). Subsequently, the patient received chemotherapy without any discomfort.

On June 14, 2023, the patient underwent enhanced chest computed tomography (CT) to evaluate the tumor during his third hospitalization. The CT scan showed that the catheter tip was located in the mediastinal fat tissue adjacent to the left brachiocephalic vein. The radiologists concluded that the tip had entered the left thymic vein (Fig. 1B and C). However, before the medical staff received a formal CT report on June 19, the patient had been discharged the previous day; thus, no further intervention on the catheter was performed. On August 1, the patient was readmitted to our hospital. Given that the previous radiograph indicated an abnormal catheter tip position, emergency AP-CXR was performed on the day of admission, which showed that the catheter tip was located at the T6 level (Fig. 1D). On August 2, the patient underwent a contrast-enhanced chest CT for reevaluation of the tumor, which indicated that the catheter tip was still within the left thymic vein (Fig. 1E and F). Nevertheless, the patient was discharged 2 days before the CT reports were received on August 6. Subsequently, telephonic follow-up revealed that the patient had discontinued further chemotherapy and had the PICC removed at a local hospital. He did not report any catheter tip-related adverse effects (Table 1).

**Table 1:** The flowchart for describing Patient 1.

Time line	Patient general information
10-Mar-2023	A 74-year-old male patient, diagnosed with esophageal cancer with pulmonary metastasis.
	Catheterization and chemotherapy (first admission to hospital)
17-Mar	***Method***: catheter was inserted via the left basilic vein under local anesthesia and ultrasound guidance.
	***Procedure***: proceeded smoothly with good blood return, effortless guide wire retraction, and no discomfort reported by the patient.
17-Mar	***Post procedure AP-CXR***: catheter tip located at sixth thoracic vertebra level, with tip appearing as a left-direction crook (Fig. 1A).
22-Mar	***Chemotherapy regime*:** cisplatin and fluorouracil were infused through catheter uneventfully, without any discomforts reported by the patient.
23-Mar	Patient discharged from hospital.
	**Discovery of catheter malposition** (third admission to hospital)
14-Jun	***Contrast-enhanced chest CT*** for tumor evaluation: an incidental discovery showed that the catheter tip was located in the mediastinal fat tissue adjacent to the left innominate vein (Fig. 1B,C).However, this finding was overlooked by the medical staff on the CT imaging.
17-Jun	***Chemotherapy regime*:** albumin-bound paclitaxel and carboplatin were infused through catheter uneventfully, without any discomforts reported by the patient.
18-Jun	***Patient discharged from hospital:*** without managing the malpositioned catheter.
19-Jun	***Formal report released by radiologist:*** radiologists concluded that the catheter tip entered into the left thymic vein.
	Intervention of catheter malposition (fourth admission to hospital)
01-Aug	***Emergency AP- CXR:*** catheter tip still located at the sixth thoracic vertebra level (Fig. 1D).
02-Aug	***Contrast-enhanced chest CT*** for reevaluation of tumor: showed the catheter tip was located within the left thymic vein, without perforating the vessel.
04-Aug	***Patient discharged from hospital:*** without managing the malpositioned catheter.
06-Aug	***Formal report released by radiologist:*** radiologists concluded that the catheter tip was still located in the left thymic vein, without perforating the vessel (Fig. 1E,F).
	**Follow-up**
8-Aug	***Catheter removal:*** the catheter was removed at a local hospital according to our telephone instructions.
16-Aug	The patient did not report significant catheter tip-related complications.

### 2.2. Patient 2

**Figure 2.**
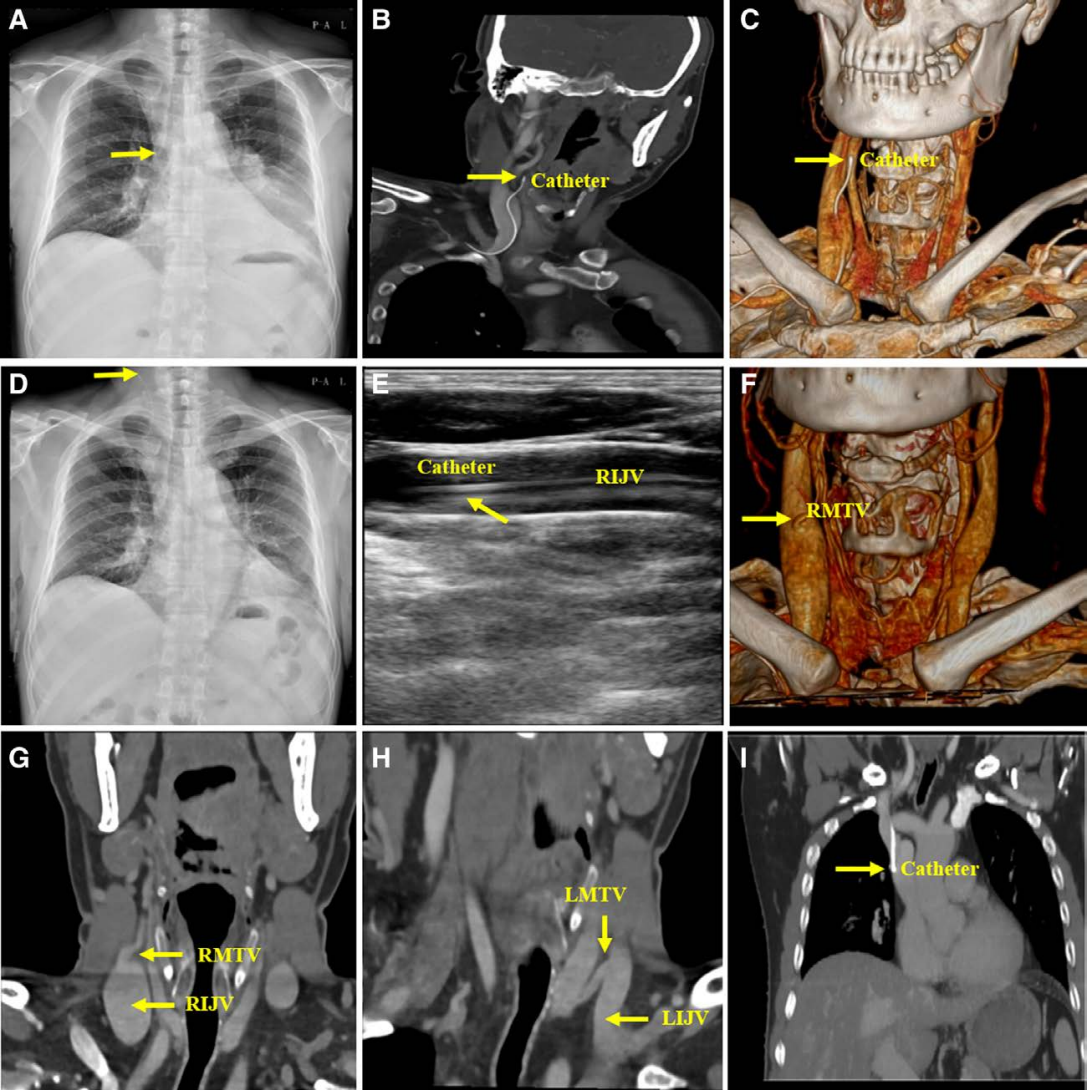
Radiographic information for Patient 2.

A 55-year-old male patient who had been diagnosed with lung cancer with bilateral cervical lymph node metastases was admitted to the oncology center for chemotherapy on May 30, 2023. On June 9, the same advanced practice nurse smoothly inserted a PICC for this patient, using the same procedure as for Patient 1. Immediately following catheterization, an AP-CXR was performed, which showed the catheter descending along the right side of the spine, with the catheter tip located at the T6 level, indicating the correct position in the superior vena cava (SVC) (Fig. 2A). On June 11, the patient received a chemotherapy regimen of pemetrexed, carboplatin, and sintilimab via the catheter without any discomfort. After the completion of chemotherapy, the patient was discharged.

The patient was readmitted to our hospital on September 22 and underwent a contrast-enhanced neck CT to assess the cervical lymph nodes on September 26. Unexpectedly, the CT scan revealed that the catheter had entered the internal jugular vein (IJV), with the catheter tip positioned outside the vascular contour of the right IJV (RIJV); however, no signs of contrast leakage were observed (Fig. 2B and C). The patient immediately underwent emergency AP-CXR, showing the catheter passing through the clavicular region to the neck, with the catheter tip oriented upwards, located at the sixth cervical vertebral level (Fig. 2D). Following vascular surgery consultation, an ultrasound examination of the IJV was conducted, revealing that the catheter tip was located in the mid-segment of the RIJV (Fig. 2E). A multidisciplinary team, including oncologists, advanced practice nurses, and vascular surgeons, reviewed the radiological examinations before and after catheterization (Fig. 2B–H), concluded that the catheter tip was within the RMTV without perforating the vessel, and deemed that it could continue to be used after repositioning the catheter tip. Subsequently, the advanced practice nurse adjusted the catheter tip under ultrasound guidance, and ultrasonography confirmed that the catheter was not within the right internal jugular vein after the adjustment. However, the patient did not undergo chest radiography, and the catheter was used for chemotherapy. The patient was readmitted on November 20 and underwent contrast-enhanced chest CT, which showed the catheter tip at the lower fourth thoracic vertebra level in the SVC (Fig. 2I). Follow-up did not reveal any patient discomfort (Table 2).

**Table 2:** The flowchart for describing Patient 2.

Time line	Patient general information
30-May-2023	A 55-year-old man with lung cancer with bilateral cervical lymph node metastasis.
	Catheterization and therapy (first admission to hospital)
09-Jun	***Catheterization**:* catheter was inserted via the right basilic vein under local anesthesia and ultrasound guidance.
	***Procedure*:** proceeded smoothly with good blood return, effortless guide wire retraction, and no discomfort reported by patient.
09-Jun	***Post procedure AP-CXR***: catheter descending along the right side of the spine, with the catheter tip located at the sixth thoracic vertebra level (Fig. 2A).
11-Jun	***Chemotherapy regime***: pemetrexed, carboplatin and sintilimab were infused through catheter uneventfully without any discomforts reported by the patient.
13-Jun	Patient discharged from hospital.
	**Discovery of catheter malposition** (third admission to hospital)
26-Sept	***Contrast-enhanced neck CT*** for evaluation of lymph node: an incidental discovery revealed that the catheter entered in the RIJV, with the catheter tip positioned outside the vascular contour of the RIJV. However, no signs of contrast leakage were observed (Fig. 2B,C).
	**Intervention of catheter malposition**
26-Sept	***Emergency AP-CXR***: catheter passing through the clavicular region to the neck, with the catheter tip oriented upwards, locating at the sixth cervical vertebrae level (Fig. 2D).
27-Sept	***Ultrasound examination***: catheter tip located in the mid-segment of the RIJV (Fig. 2E).
27-Sept	***Multidisciplinary team consultation***: oncologists, advanced practice nurse and vascular surgeons reviewed the radiographic imaging before and after catheterization (Fig. 2B–H), concluding that the catheter tip was located within the RMTV, without perforating the vessel.
27-Sept	***Management***: an advanced practice nurse adjusted the catheter tip under ultrasound guidance and using ultrasound to confirm that the catheter was not within the RIJV post-adjustment. No further chest radiography was performed to verify the position of the catheter tip.
27-Sept	***Chemotherapy regime***: pemetrexed, carboplatin and sintilimab were infused through catheter uneventfully without any discomforts reported by the patient.
01-Oct	Patient discharged from hospital.
	**Follow-up**
23-Nov	***Contrast-enhanced chest CT*** for tumor evaluation: showed the catheter tip located at the lower fourth thoracic vertebra in the superior vena cava (Fig. 2I).
24-Nov	***Chemotherapy regime***: pemetrexed, carboplatin and sintilimab were infused through catheter uneventfully without any discomforts reported by patient.
25-Nov	Patient discharged from hospital.

## 3. Discussion

The thymic vein is located posterior to the thymus and exhibits significant anatomical variability, ranging from 1 to 3 in number, with the majority draining into the left brachiocephalic vein. The thymic vein has a diameter ranging from 1.0 to 3.4 mm, with an average diameter of 2.0 mm, which is difficult to visualize on non-enhanced imaging, requiring contrast-enhanced CT for visualization.^[3]^ Under normal circumstances, a catheter located in the SVC should be oriented vertically downward and parallel to its longitudinal axis. Post hoc analysis did not identify high-risk factors for catheter malpositioning in the patient 1, typically associated with venous dilation subsequent to vena cava obstruction syndrome, heart failure, or obstruction of the superior-inferior vena cava system circulation. After reviewing the AP-CXR image, we observed that the catheter tip was located at the T6 level, not vertically downward, with the tip appearing as a left-direction crook (Fig. 1A). An atypical shape of the catheter tip can be recognized as a subtle indication of catheter malposition. The AP-CXR alone cannot definitively determine the catheter position.^[4]^ In the process of evaluating the tumor, the patient underwent contrast-enhanced CT, and subsequently, the catheter tip was confirmed to be mispositioned into the left thymic vein. However, the medical staff repeatedly neglected all the abnormal signs on CT imaging for various reasons, causing the malpositioned catheter not to be properly managed in time. There have been 2 reported cases of centrally inserted venous catheter malpositioning into the thymic vein before our report; one case involved a centrally inserted venous catheter inserted via the RIJV, which was found to be malpositioned into the right thymic vein during the operation of mitral valve replacement.^[5]^ The other case was only suspected to have been mispositioned into the thymic vein via the RIJV because the case only received an AP-CXR examination,^[6]^ making it difficult to ascertain whether the catheter was actually inside the thymic vein. Therefore, to our knowledge, the present case is most likely the first of PICC malposition in the thymic vein.

The second case presented here was a secondary PICC malpositioning in the middle thyroid vein. The thyroid gland is richly vascularized and drained by 3 pairs of veins: the superior, middle, and inferior thyroid veins, with the latter draining into the brachiocephalic vein and the others draining into the IJV.^[7]^ Therefore, any catheter located in the IJV could enter the venous branches. Ng et al reported that a PICC malpositioned in the inferior thyroid vein resulted in fatal complications, and the patient had to undergo a second surgery to manage the malpositioned catheter.^[8]^ As in the patient 1, the abnormal catheter position was incidentally revealed by contrast-enhanced CT. Afterward, AP-CXR and vascular ultrasonography were performed on the neck. Based on all imaging examinations, the multidisciplinary team concluded that the catheter tip was located within the RMTV without contrast leakage, indicating that the catheter had not perforated the vessel. Because the catheter was still in use, we decided to keep it in the body if it could be repositioned into the SVC. In the post hoc analysis, the patient reported having repeated blood backflow in the catheter, which caused him to visit the outpatient clinic to deal with the blood backflow. In our clinical experience, repeated blood backflow in the catheter is often associated with a catheter tip located in a non-central vein, such as the brachiocephalic vein or IJV. In addition, the patient had lung cancer and experienced long-term hemoptysis and coughing before and after catheterization, which could have caused significant changes in thoracic pressure and induced secondary malpositioning. We also observed the anatomical variation of the left middle thyroid vein and RMTV on the CT, which showed that the connection of the RMTV and IJV was straight, while the connection of the left middle thyroid vein and IJV was Z-shaped (Fig. 2G and H). This preexisting variation of the RMTV is an anatomical risk factor that further induces mispositioning. Compared with the SVC, the RMTV has thin and fragile venous walls; neck movements can cause mechanical damage, and drug infusion can cause chemical damage to the vessel or even perforation, leading to significant hemorrhage, tracheal compression, respiratory distress, asphyxiation, or even death.

The management of these 2 cases of catheter malposition had deficiencies. In the first case, the radiographic report was released the day after the patient had been discharged twice in a row, preventing the catheter from being handled in a timely manner during the hospital stay. Fortunately, the patient had the catheter removed at a local hospital after discharge, which avoided potential life-threatening complications such as breast abscesses, seizures, or even death.^[4,9,10]^ In the second case, after adjusting the catheter position, we did not perform a radiographic examination to verify the catheter tip position before using it for chemotherapy infusion. Instead, confirmation that the catheter tip was within the SVC was delayed until the patient’s next admission, when he underwent CT.

## 4. Conclusion

Several conclusions can be drawn from these 2 rare cases of PICC malpositioning. First, the principle of “verify tip before using catheter” must be strictly adhered to. Second, real-time imaging guidance should be utilized for catheterization as much as possible, and intracavitary electrocardiogram-guided technology is recommended. Third, attention should be paid to abnormal signs of catheter malposition, not only signs on radiographic imaging but also newly occurring clinical signs, such as abnormal blood return in the catheter or catheter dysfunction. Fourth, catheter monitoring: patients with long-term catheters should undergo regular radiographic examinations to check the catheter tip and detect secondary malposition in time. Fifth, a comprehensive review of chest imaging is essential. Because of the strain on medical resources, hospital stays are often shortened, leading to radiographic reports being available only after patient discharge. Therefore, clinical staff should actively assess the catheter tip position in the imaging system when evaluating a tumor rather than waiting for a radiologist’s report. Particularly in cases where catheter tip malposition is highly suspected, proactive assessment or direct communication with radiologists can lead to early diagnosis of catheter malposition and ensure timely and proper management of the catheter. Finally, if a mispositioned catheter must be removed, it is necessary to ensure that the blood vessel has not been perforated before catheter removal to avoid fatal bleeding in the mediastinum and neck.

## Author contributions

**Conceptualization:** Xuemei Li, Xiaoxia Zhang.

**Formal analysis:** Xuemei Li.

**Methodology:** Xuemei Li, Xiaoxia Zhang.

**Resources:** Chang Liu.

**Supervision:** Xiaoxia Zhang.

**Writing – original draft:** Xuemei Li.

**Writing – review & editing:** Chang Liu, Hongxiu Chen, Zhoupeng Wu, Danmei Liang, Xiaoxia Zhang.
